# Mitogenome evolution in ladybirds: Potential association with dietary adaptation

**DOI:** 10.1002/ece3.5971

**Published:** 2020-01-02

**Authors:** Ming‐Long Yuan, Li‐Jun Zhang, Qi‐Lin Zhang, Li Zhang, Min Li, Xiao‐Tong Wang, Run‐Qiu Feng, Pei‐An Tang

**Affiliations:** ^1^ State Key Laboratory of Grassland Agro‐Ecosystems Key Laboratory of Grassland Livestock Industry Innovation, Ministry of Agriculture and Rural Affairs Engineering Research Center of Grassland Industry, Ministry of Education College of Pastoral Agriculture Science and Technology Lanzhou University Lanzhou China; ^2^ Faculty of Life Science and Technology Kunming University of Science and Technology Kunming China; ^3^ Collaborative Innovation Center for Modern Grain Circulation and Safety College of Food Science and Engineering Nanjing University of Finance and Economics Nanjing China

**Keywords:** diet evolution, mitochondrial DNA, molecular phylogeny, negative selection, positive selection

## Abstract

Dietary shifts can alter the relative availability of different nutrients and are therefore associated with metabolic adaptation in animals. The Coccinellidae (ladybirds) exhibits three major types of feeding habits and provides a useful model to study the effects of dietary changes on the evolution of mitogenomes, which encode proteins directly involved in energy metabolism. Here, mitogenomes of three coccinellid species were newly sequenced. These data were combined with other ten previously sequenced coccinellid mitogenomes to explore the relationship between mitogenome evolution and diets. Our results indicate that mitogenomic data can be effectively used to resolve phylogenetic relationships of Coccinellidae. Strong codon usage bias in coccinellid mitogenomes was predominantly determined by nucleotide composition. The 13 mitochondrial protein‐coding genes (PCGs) globally evolved under negative constraints, with some PCGs showing a stronger purifying selection. Six PCGs (*nad3*, *nad4L,* and *nad5* from Complex I; *cox1* and *cox3* from Complex IV; and *atp6* from Complex V) displayed signs of positive selection. Of these, adaptive changes in *cox3* were potentially associated with metabolic differences resulting from dietary shifts in Coccinellidae. Our results provide insights into the adaptive evolution of coccinellid mitogenomes in response to both dietary shifts and other life history traits.

## Graphical abstract

We sequenced for the first time three coccinellid mitogenomes. Six mitochondrial PCGs displayed signs of positive selection. Adaptive evolution of coccinellid mitogenomes is potentially associated with both dietary shifts and other life history traits.

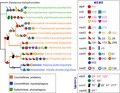

## 1 INTRODUCTION

Coccinellidae (ladybirds), comprising over 6,000 species in roughly 360 genera, are the largest lineage in the superfamily Coccinelloidea (formerly known as the Cerylonid Series within the superfamily Cucujoidea (Hunt et al., 2007; Robertson et al., 2015)). Traditionally, the classification system of Coccinellidae includes six or seven subfamilies, although only two subfamilies (Coccinellinae and Microweiseinae) were found to be truly monophyletic based on molecular and morphological data (Giorgi et al., 2009; Magro, Lecompte, Magne, Hemptinne, & Crouau‐Roy, 2010; Seago, Giorgi, Li, & Slipinski, 2011). Ladybirds show high diversity in ecology, morphology, behavior, and diet. Three major types of feeding habits are generally recognized for coccinellids: predatory (most coccinellid species), phytophagous (those feeding on plants, e.g., *Henosepilachna vigintioctopunctata*) and mycetophagous (those feeding on fungi, e.g., *Halyzia sedecimguttata*). Dietary shifts in ladybirds have been associated with changes in physiological metabolism and ecological adaptation (Escalona, Zwick, et al., 2017; Giorgi et al., 2009; Li, Pan, Clercq, Ślipiński, & Pang, 2016). However, the evolutionary processes and molecular mechanisms of these shifts largely remain unclear.

Mitochondria supply up to 95% of the energy needs of eukaryotic cells through oxidative phosphorylation (OXPHOS), carried out by five multi‐subunit OXPHOS complexes. Four complexes (I, III, IV, and V) have subunits encoded by genes in both the nuclear and mitochondrial genomes, whereas one (II) is encoded exclusively in the nuclear genome. Given the functional importance of OXPHOS, variations in these genes can directly influence metabolic performance (Escalona, Weadick, & Antunes, 2017; da Fonseca, Johnson, O'Brien, Ramos, & Antunes, 2008; Hood et al., 2018; Li et al., 2017; Scott, Guo, & Dawson, 2018). Numerous studies have shown that non‐neutral mutations in mitochondrial‐encoded protein‐coding genes (PCGs) are associated with adaptations of animals to different environments (Almeida, Maldonado, Vasconcelos, & Antunes, 2015; da Fonseca et al., 2008; Luo, Yang, & Gao, 2013; Pfenninger et al., 2014; Scott et al., 2018, 2011; Yuan et al., 2018; Zhang, Yang, & Zhang, 2019). Dietary shifts can alter the relative availability of different nutrients and are therefore associated with metabolic adaptation in animals (Babbitt, Warner, Fedrigo, Wall, & Wray, 2010; Ballard & Youngson, 2015; Li et al., 2016; Pontremolil et al., 2015; Ravenscraft & Boggs, 2016). Here, we propose that differences in diets among ladybirds should cause adaptive evolution of their mitogenomes.

In this study, three previously unsequenced coccinellid mitogenomes, representing three coccinellid groups with different diets, were sequenced. These data were then used to correlate their different diets with differences in the evolution of their mitochondrial genes. Our results provided the evidence that dietary shifts associated with metabolic differences potentially contribute to mitogenome evolution in Coccinellidae.

## 2 MATERIALS AND METHODS

### 2.1 Sampling, DNA extraction, and sequencing

Samples of three coccinellid species, *Coccinella transversoguttata* (predatory), *H. vigintioctopunctata* (phytophagous), and *Vibidia duodecimguttata* (mycetophagous), were collected from China in 2014 and 2015 (Table S1). All samples were initially preserved in 100% ethanol in the field and transferred to −20°C until used for DNA extraction. Samples were identified by M.L. Yuan and Q.L. Zhang based on morphological characteristics. Voucher specimens were deposited in College of Pastoral Agricultural Science and Technology, Lanzhou University, Lanzhou, P. R. China. For each species, the total genomic DNA was extracted from thorax muscle of a single specimen using the OMEGA Insect DNA Kit (OMEGA). Mitogenome sequences of the three ladybirds were amplified with overlapping fragments using universal insect mitochondrial primers (Simon, Buckley, Frati, Stewart, & Beckenbach, 2006) and species‐specific primer pairs (Table S2). For *C. transversoguttata*, the species‐specific primers were designed from sequenced fragments, whereas species‐specific primers for the other two ladybirds were designed based on unigene records of their respective mitochondrial transcriptomes. PCR reactions were performed with LA Taq (TaKaRa), and all PCR fragments were sequenced in both directions on an ABI3730 automated sequencer (Applied Biosystems), as described previously (Yuan, Zhang, Guo, Wang, & Shen, 2015a, 2015b).

### 2.2 Sequence annotation and analysis

All the 13 PCGs were determined using ORF Finder online at the NCBI website, and the two rRNAs were identified by aligning with that of other coccinellid species, as described in our previous studies (Yuan et al., 2015a, 2015b). The 22 tRNAs were predicted using tRNAscan‐SE 1.21 (Lowe & Eddy, 1997). The three coccinellid mitogenomes have been deposited in GenBank (accession numbers MG584726–28). Nucleotide composition and the relative synonymous codon frequencies were calculated with MEGA 6.06 (Tamura, Stecher, Peterson, Filipski, & Kumar, 2013). The effective number of codons (ENC) for each species was determined with DnaSP 5.0 (Librado & Rozas, 2009). The ENC is used to measure how far the codon usage of a gene departs from equal, unbiased usage of synonymous codons (Wright, 1990). ENC values may range from 20 (extreme codon bias, only one codon is used for each amino acid) to 61 (no codon bias, all synonymous codons are used equally). Generally, ENC values of 35 or less are considered biased (Wright, 1990). To examine the influence of GC content on codon usage, the relationship between ENC and GC content of the third codon positions (GC3) was plotted. The ENC* value for each species was also calculated using the formula: ENC* = 2 + GC3 + (29/[(GC3)^2^ + (1 − GC3)^2^]) (Wright, 1990). Strand asymmetry was calculated using the formulas: AT‐skew = (A − T)/(A + T) and GC‐skew = (G − C)/(G + C) (Perna & Kocher, 1995).

### 2.3 Phylogenetic analysis

Phylogenetic analyses based on the nucleotide sequences of 13 PCGs were conducted with Bayesian inference (BI) and maximum‐likelihood (ML) methods available on the CIPRES Science Gateway 3.3 (Miller, Pfeiffer, & Schwartz, 2010). Each PCG was used to generate multiple codon‐based alignments with MAFFT, as implemented in the TranslatorX online server (Abascal, Zardoya, & Telford, 2010). Poorly aligned regions were removed using GBlocks within the TranslatorX under default settings. Alignments of individual genes were then concatenated as a combined dataset (P123) with 11,178 nucleotides. Another dataset (P12) was also generated, comprising of the first and second positions of 13 PCGs, including 7,452 nucleotides. We employed DAMBE 5.3.74 (Xia, 2013) to perform a test of substitution saturation for the P123 and P12 datasets. The best partitioning schemes and corresponding nucleotide substitution models for the two datasets were determined by using PartitionFinder 1.1.1 (Lanfear, Calcott, Ho, & Guindon, 2012), as previously described (Yuan et al., 2015a, 2016).

Bayesian analysis was conducted with MrBayes 3.2.3 (Ronquist et al., 2012). Two independent runs with four chains (three heated and one cold) were performed simultaneously for 1 × 10^7^ generations. Each set was sampled every 100 generations. Stationarity was assessed by estimated sample size and potential scale reduction factor values (Ronquist et al., 2012). We discarded the first 25% samples as burn‐in and calculated posterior probabilities (PP) using the remaining samples in a 50% majority‐rule consensus tree. ML analysis was performed with RAxML‐HPC2 on XSEDE 8.2.10 (Stamatakis, 2014) using the GTRGAMMAI model. The node reliability was evaluated by conducting rapid bootstrapping (BS) with 1,000 iterations.

### 2.4 Selective pressure analysis

To evaluate the influence of natural selection on the evolution of 13 PCGs in ladybirds, the number of synonymous substitutions per synonymous sites (*d*_S_) and the number of nonsynonymous substitutions per nonsynonymous sites (*d*_N_) were calculated. Generally, sites are considered neutral if *d*_N_/*d*_S_ (*ω*) = 1, under negative/purifying selection if *d*_N_/*d*_S_ (*ω*) < 1, and under positive/diversifying selection if *ω* > 1 (Anisimova & Kosiol, 2009). The codeml program in PAML 4.7 (Yang, 2007) was used to analyze the selective pressures for each PCG under three types of models (random site, branch‐specific, and branch‐site) using a maximum‐likelihood approach (Yang, 2007). Six M‐series models were used to account for the *ω* variation among sites: M0 (one ratio), M1a (*ω* ≤ 1, nearly neutral), M2a (*ω* ≤ 1 and *ω* > 1; positive selection), M3 (discrete), M7 (*β*, a discrete distribution with 10 site classes to model values of *ω* between 0 and 1), and M8 (*β* and *ω* > 1). To determine which model fit the data significantly better, a log‐likelihood ratio test (LRT) was conducted by comparing the following nested models: M0‐M3, M1a‐M2a, and M7‐M8. Positively selected sites were detected when *ω* > 1 and the LRT was significant (*p* < .05).

To detect if there were significant changes in selective pressures among ladybirds with different diets (predatory, phytophagous, and mycetophagous), a branch‐specific model was applied. This model assumes four *ω* values: one for all the deep branches and three for each of the external branches of three dietary ladybirds. A branch‐site model was also used to investigate whether some sites have undergone positive selection in a foreground branch among the phylogenetic tree. In this analysis, each of the 25 foreground branches (branches a–x and s + v, predatory ladybirds; Figure S1) was examined for possible signals. In branch‐specific and branch‐site analyses, the phylogenetic tree (unrooted) inferred from the P123 dataset was set as the guide tree (Figure S1) and LRTs were used to determine which model better fits the data (*p* < .05). Positive selection was inferred when *ω* > 1 and the LRT was significant (*p* < .05) (Yang, 2007). The Bayes empirical Bayes (BEB) method was used to calculate posterior probabilities for site classes to determine which codon positions have experienced positive selection (*ω* > 1) (Zhang, Nielsen, & Yang, 2005).

Two models on the Datamonkey web server (http://www.datamonkey.org/) were used to analyze selective pressures: the fixed effects likelihood mode (FEL, site‐by‐site analysis) (Pond & Frost, 2005) and the mixed effects model of evolution (MEME, branch‐site analysis) (Murrell et al., 2012). In all analyses, best‐fit substitution models were used for each PCG, directly evaluated on the Datamonkey web server. Positively selected sites were inferred at the *p* < .05 significance level.

## 3 RESULTS

### 3.1 General features of three newly sequenced mitochondrial genomes

We obtained the complete mitogenomes of *C. transversoguttata* and *H. vigintioctopunctata*, and the nearly complete mitogenome of *V. duodecimguttata* (Table 1). The *H. vigintioctopunctata* genome encoded all typical 37 mitochondrial genes, whereas *C. transversoguttata* contained 36 genes due to the lack of *trnI* (Table S3). For *V. duodecimguttata*, the region that generally contains three tRNA genes (*trnI*, *trnQ* and *trnM*) and a putative control region in insect mitogenomes could not be sequenced. Several gene overlaps were consistently presented in the three ladybirds, with three large overlaps in *trnC*/*trnY* (−8 bp), *atp8*/*atp6* (−7 bp), and *nad4*/*nad4L* (−7 bp) (Table S3). The intergenic spacers of the three ladybirds ranged from 22 bp (*C. transversoguttata*) to 175 bp (*V. duodecimguttata*), spread over five to six positions (Table S3). The majority of spacer regions were short (≤2 bp), but a large intergenic spacer between *trnS2* and *nad1* was consistently found in the three ladybird species (17–30 bp). In addition, a larger intergenic spacer was present in *trnI*/*trnQ* (39 bp) of *H. vigintioctopunctata* and in *trnK*/*trnD* (140 bp) of *V. duodecimguttata*.

**Table 1:** Species used in this study

Family/Subfamily	Species	GenBank nos.	Size (bp)	A + T%	*trnI*	AT‐skew	GC‐skew	Reference
Bothrideridae	*Dastarcus helophoroides*	NC_024271	15,878	79.1	√	0.040	−0.193	Zhang et al., 2015
Coccinellidae								
Coccinellinae								
Coccinellini	*Coccinella septempunctata*	JQ321839	18,965	77.0	√	0.012	−0.086	Kim, Wan, & Kim, 2012
	*Coccinella transversoguttata*	MG584726	15,806	77.7	×	0.034	−0.170	This study
	*Anisosticta novemdecimpunctata*	KT876880 *	15,289	78.9	–	0.054	−0.196	Linard, Arribas, Andújar, Crampton‐Platt, & Vogler, 2016
	*Calvia championorum*	KX132085 *	17,575	78.2	√	0.059	−0.132	unpublished
	*Cheilomenes sexmaculata*	KM244706	17,192	78.1	√	0.048	−0.195	Tang et al., 2014
	*Cycloneda sanguinea*	KU877170 *	18,715	79.3	√	0.054	−0.169	unpublished
	*Harmonia axyridis*	KR108208 *	16,387	78.7	–	0.030	−0.225	Niu, Zhu, Wang, & Wei, 2016
	*Propylea japonica*	KM244660 *	15,027	79.1	–	0.054	−0.136	Tang et al., 2014
Halyziini	*Halyzia sedecimguttata*	KT780652 *	15,766	78.1	–	0.060	−0.188	unpublished
	*Vibidia duodecimguttata*	MG584728 *	14,431	77.3	–	0.062	−0.185	This study
Epilachninae								
Epilachnini	*Henosepilachna pusillanima*	NC_023469	16,216	78.2	√	0.028	−0.199	Behere, Firake, Tay, Azad Thakur, & Ngachan, 2016
	*Henosepilachna vigintioctopunctata*	MG584727	17,057	79.2	√	0.022	−0.203	This study
	*Subcoccinella vigintiquatuorpunctata*	KT780695 *	14,645	76.2	√	0.027	−0.219	unpublished

Note

√, *trnI* is present. ×, *trnI* is absent. –, it is uncertain whether *trnI* is present due to an incomplete sequencing.

* Incomplete mitogenome.

All the PCGs began with an ATN codon, except for *cox2* (TTG) in *H. vigintioctopunctata*, *cox1* (AAT) in both *V. duodecimguttata* and *C. transversoguttata* (Table S3). All tRNAs in the three ladybird species possessed a canonical cloverleaf secondary structure, except for *trnS1* (AGN) that lacked the dihydrouridine (DHU) arm (Figure S2). The sequences and structures of anticodon arms and DHU stems of all 22 tRNAs were highly conversed across the 13 ladybirds, and most variations (substitutions and indels) were present in DHU loops, pseudouridine (TψC) arms, and variable loops (Figure S2).

### 3.2 Nucleotide composition and codon usage

The newly sequenced mitogenomes of three coccinellid species presented a similar nucleotide composition, that is, high A + T content (77.3%–79.2%), slight positive AT skews (0.022–0.062), and moderate negative GC skews (−0.170 to 0.302) (Table 1). This nucleotide composition bias was also reflected in the codon usage pattern, that is, the fourfold and twofold degenerate codons used more As and Ts than Gs and Cs (Tables [Link], [Link], [Link]). Among 62 amino acid encoding codons of invertebrate mitochondrial code, some GC‐rich codons were never utilized, such as CCG in *H. vigintioctopunctata* and GTC in *C. transversoguttata* (Tables S4 and S6).

**Figure 1:**
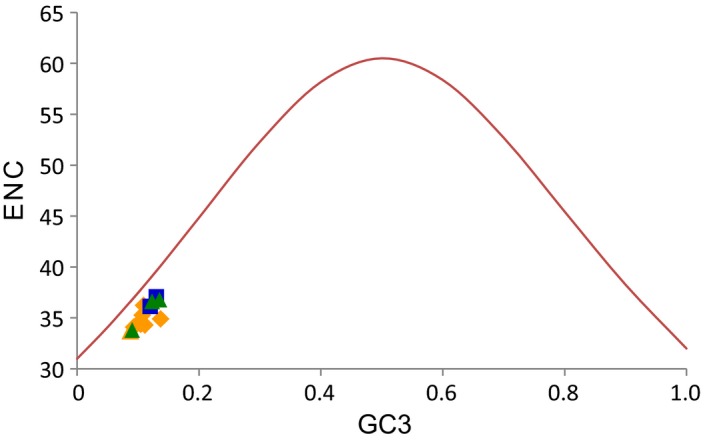
The correlation between effective number of codons (ENC) and G + C content of the third codon positions (GC3) for 13 coccinellid species. The solid line represents the relationship between the ENC* and the GC3 content. The symbols under the red line represent diets and the colors match those in the Figures 2 and 4

To further investigate the codon usage bias, ENC and the G + C content were analyzed for all 13 ladybird mitogenomes. The average ENC value of the mycetophagous ladybirds was the highest (mean = 36.6, *SD* = 0.66), but not significantly higher than those of the predatory (mean = 34.6, *SD* = 0.78) or phytophagous species (mean = 35.7, *SD* = 1.67) (Table S7). This was also true for the G + C content of all positions (GCa) and the third positions (GC3) among ladybirds with different diets (*p* > .05, Table S7). A significant positive correlation was found between ENC and GCa (*R* ^2^ = .69, *p* < .001), and also between ENC and GC3 (*R* ^2^ = .59, *p* < .001). The actual ENC values for all ladybirds were just below the ENC* curve (Figure 1).

### 3.3 Mitochondrial phylogeny of Coccinellidae

**Figure 2:**
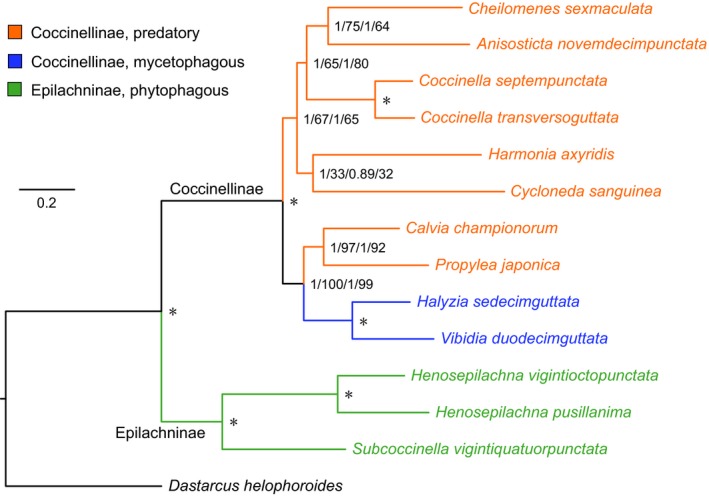
Phylogenetic relationships among 13 Coccinellidae species inferred from mitochondrial genome sequences. Three species from Epilachninae and ten species from Coccinellinae are included in this phylogenetic analysis. Numbers from left to right are Bayesian posterior probabilities (PP) and ML bootstrap (BS) values of each of the two datasets (PCG123 and PCG12). Asterisk (*) indicates PP = 1.0 and BS = 100

Tests of substitution saturation for the P123 and P12 datasets showed a substantial substitution saturation in the third positions of 13 PCGs (Table S8). BI and ML analyses were performed using two datasets (PCG123 and PCG12) under the best partitioning schemes and corresponding nucleotide substitution models, as determined by PartitionFinder (Table S9). All phylogenetic analyses obtained the same tree topology (Figure 2). The monophyly of each of the two subfamilies (Coccidulinae and Epilachninae) were highly supported by the data (PP = 1, BS = 100; Figure 2). Within Coccinellinae, Halyziini (mycetophagous ladybirds) was supported as a monophyletic group, while Coccinellini (predatory ladybirds) was a paraphyletic group in which *Calvia championorum* and *Propylea japonica* clustered with two Halyziini species. For the remaining predatory ladybirds, a phylogeny of (*Cycloneda* + *Harmonia*) + (*Coccinella* + (*Cheilomenes* + *Anisosticta*)) was found (Figure 2).

### 3.4 Positive selection test of mitochondrial protein‐coding genes in Coccinellidae

**Figure 3:**
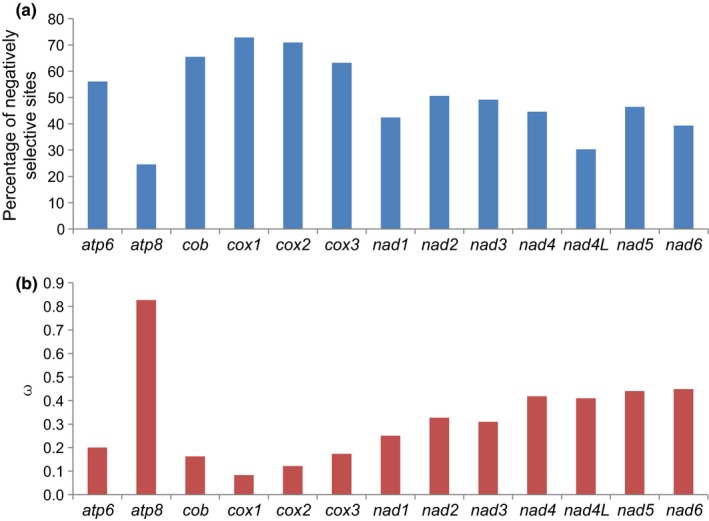
Results of analysis for negative selection using FEL site‐by‐site analyses. (a) Percentage of sites subject to negative selection in each of 13 protein‐coding genes in Coccinellidae. (b) The ratios (*ω*) of nonsynonymous substitutions to synonymous substitutions for each of 13 protein‐coding genes in Coccinellidae

For random site model analyses in codeml, LRT tests for each mitochondrial PCG showed that the M3 model fitted better than the M0 model (*p* < .05) (Table S10), indicating that *ω* differed among sites in all the 13 PCGs. LRT values for both M1a–M2a and M7–M8 were not significant (*p* < .05) (Table S10), indicating an absence of positively selected sites. All 13 coccinellid PCGs were thus found to be globally evolving under negative constraints. Further FEL site‐by‐site analyses were performed to detect which codons were under negative selection. The results showed that *cox1* presented the highest percentage of codons under negative selection (72.8%), followed by *cox2* (70.9%), *cob* (65.4%) and *cox3* (63.2%) (Figure 3a). These four genes also showed a smaller *ω* (0.08–0.17), indicating that they were under stronger purifying selection than the remaining nine genes (Figure 3b). These nine genes showed a relaxed *ω* value, ranging from 0.20 in *atp6* to 0.83 in *atp8*.

In branch‐specific analyses, a four‐ratio model allowing different *ω* for the internal braches and each of terminal branches leading to different feeding habits showed significantly better results than a one‐ratio model (*p* < .05) (Table 2). For the concatenated 13PCGs, the estimated *ω* for phytophagous ladybirds was higher than that for predatory and mycetophagous ladybirds separately. For individual genes, four (*cob*, *cox1*, *nad2,* and *nad4*) showed a higher *ω* in phytophagous ladybirds, four (*cox2*, *nad1*, *nad5,* and *nad6*) showed a higher *ω* in predatory ladybirds, and the remaining five showed a higher *ω* in mycetophagous ladybirds (Table 2). These results implied that ladybirds with different diets were evolving under different selective pressures and accumulated more nonsynonymous substitutions in some mitochondrial PCGs.

**Table 2:** Estimation of *ω* (*d*_N_/*d*_S_) values for the 13 mitochondrial protein‐coding genes in 13 ladybirds

Gene	Estimated *ω* values			2Δ*ℓ*	*p*
	Mycetophagous	Phytophagous	Predatory		
*atp6*	0.024	0.023	0.017	18.4	<.001
*atp8*	0.082	0.002	0.012	4.0	.045
*cob*	0.013	0.023	0.012	30.9	<.001
*cox1*	0.007	0.010	0.010	65.5	<.001
*cox2*	0.003	0.012	0.017	40.8	<.001
*cox3*	0.032	0.028	0.024	11.9	.001
*nad1*	0.007	0.014	0.017	19.6	<.001
*nad2*	0.011	0.022	0.013	15.3	<.001
*nad3*	0.029	0.015	0.016	4.0	.046
*nad4*	0.009	0.018	0.012	15.3	<.001
*nad4L*	0.033	0.008	0.023	9.0	.003
*nad5*	0.013	0.013	0.014	46.7	<.001
*nad6*	0.012	0.004	0.013	6.2	.013
13 PCGs	0.017	0.020	0.019	273.0	<.001

**Figure 4:**
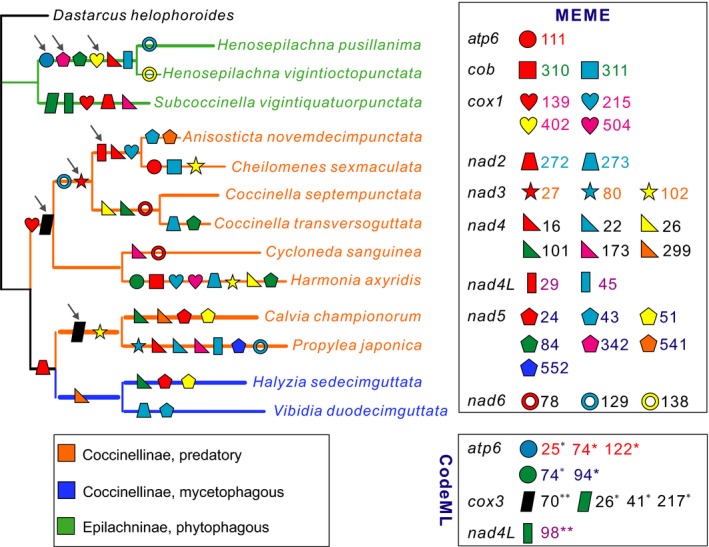
Results of analysis for positive selection using the codeml in PAML and MEME in Datamonkey. Thirteen coccinellids from two subfamilies (Epilachninae and Coccinellinae) were used in positive selection analyses. An arrow indicates potential sites of positive selection. The sites from the same genes are indicated by the same colors. Different figure shapes represent different genes in MEME or CodeML

In the branch‐site model analyses, when branches leading to the predatory ladybirds were set as the forebranches (branches s and v in Figure S1), signatures of positive selection were detected in one codon of *cox3* (Figure 4, Table S11). No significant evidence of positive selection was found in branches leading to phytophagous (branch n in Figure S1) or mycetophagous (branch x in Figure S1) ladybirds. However, three codons of *atp6* showed positive selection signals in the common ancestral branch (branch m in Figure S1) of two *Henosepilachna* species (Figure 4). Although six codons of three PCGs (*atp6*, *cox3,* and *nad4L*) were also identified as candidate sites under positive selection, these sites affected a single tip lineage, indicating that these likely were mildly deleterious mutations accumulated by relaxed purifying selection (Figure 4, Table S11). The MEME analyses identified 30 codon sites of nine PCGs with evidence of episodic diversifying selection in nine genes (Figure 4). Nine sites had a substitution affecting a single tip lineage, whereas 17 sites presented substitutions whose effects were dispersed throughout whole tree. The remaining four codons of four PCGs (*cox1*, *nad3*, *nad4L*, and *nad5*) affected more than one sequence and represented potential candidates for positive selection.

## 4 DISCUSSION

### 4.1 Characteristics of mitochondrial genomes in Coccinellidae

Metazoan mitogenomes typically encode 37 genes: 13 PCGs, two rRNAs, and 22 tRNAs. Generally, gene content in each mitogenome is highly conserved in most animals. However, variation in gene content has been frequently reported in animal mitogenomes, including loss and/or gain of PCGs, rRNAs, and/or tRNAs (Gissi, Iannelli, & Pesole, 2008; Lavrov & Pett, 2016). The tRNA gene *trnI* was not detected in the *C. transversoguttata* mitogenome. Initially, it was suspected that the loss of *trnI* may be due to incomplete sequencing, because *trnI* is generally adjacent to the control region where high A + T content, tandem repetitions and stable stem‐loop structures can disrupt PCR and sequencing reactions. Therefore, PCR amplification and sequencing were repeated, confirming the accuracy of the original sequencing results. Interestingly, *trnI* was present in the mitogenome of the congeneric *C. septempunctata*. Similarly, *trnI* was also detected in six other ladybirds, but it was difficult to determine whether this tRNA was lost or present in the remaining five ladybirds (Table 1). Therefore, the loss of *trnI* seemed not to be directly associated with dietary shifts. The lack of *trnI* has also been reported in other insects, such as weevils (Haran, Timmermans, & Vogler, 2013; Nan, Wei, & He, 2016; Song, Sheffield, Cameron, Miller, & Whiting, 2010), whiteflies (Thao, Baumann, & Baumann, 2004), and true bugs (Dai, Li, & Jiang, 2012; Li et al., 2012). It is thought that the loss of a tRNAs is compensated by the import of tRNAs from the cytoplasm (Duchêne, Pujol, & Maréchal‐Drouard, 2009). Further investigations by sequencing more ladybird species from other subfamilies and functional experiments will be needed to understand *trnI* evolution in Coccinellidae.

Generally, the insect mitogenome is highly compact, with several highly conserved gene overlaps. However, several noncoding regions were found in the coccinellid mitogenomes studied here. A large noncoding region between *trnS2* and *nad1* was consistently present in all ladybirds, as observed in other insects (Beckenbach, 2012; Cameron & Whiting, 2008). These sequences are generally conserved and have been proposed as the binding site of a transcription termination factor (DmTTF) (Roberti, Polosa, & Bruni, 2003). In addition, a larger intergenic spacer was presented in *trnI*/*trnQ* (39 bp) of *H. vigintioctopunctata* and in *trnK*/*trnD* (140 bp) of *V. duodecimguttata*. To our knowledge, such large intergenic spacers at the two locations have rarely been observed in other coleopteran species. However, these intergenic spacers were not always detected in all sequenced ladybirds, indicating they were likely species‐specific. Further studies including more ladybird species are required to better understand the origin of noncoding regions and their function in mitogenomes.

Changes in an organism's diet could have the potential to alter the nucleotide composition and codon usage of the genome (Seward & Kelly, 2016). A strong bias in nucleotide composition and codon usage was found in coccinellid mitogenomes, but there was no significant difference among the three coccinellid groups with different diets. A significant positive correlation was found between ENC and the G + C content, which was consistent with prevailing neutral mutational theories positing that genomic G + C content was the most significant factor in determining codon bias among organisms (Hershberg & Petrov, 2008; Plotkin & Kudla, 2011). On the other hand, the codon bias in coccinellid mitogenomes was likely affected by other selective pressures (e.g., generation time, body size, and habitat), as indicated by the comparison of actual ENC and ENC* (Figure 1).

### 4.2 Mitochondrial phylogeny of Coccinellidae

Over the last two decades, mitogenome sequences have been used in studies of coleopteran phylogeny at various taxonomic levels (Liu et al., 2018; López‐López & Vogler, 2017; Timmermans et al., 2016; Yuan et al., 2016). Our results, based on two mitogenomic datasets (P123 and P12) and two analytical methods (BI and ML), generated the same tree topology (Figure 2). The phylogenetic relationships were largely consistent with that previously determined (Escalona, Zwick, et al., 2017; Magro et al., 2010), indicating that mitogenomic sequences provide useful data for resolving phylogenetic relationships in Coccinellidae. Coccinellinae was strongly showed to be a monophyletic group, supporting the traditional taxonomic views (Magro et al., 2010). The monophyly of Epilachninae was also supported by this study, although this subfamily has been recognized as invalid in several previous studies (Giorgi et al., 2009; Magro et al., 2010; Seago et al., 2011). Two mycetophagous ladybirds (Halyziini) were found to be mixed with Coccinellini species, rendering Coccinellini paraphyletic; however, the former Halyziini has been proposed to be a subtribe within Coccinellini by previous studies based on morphological and molecular data (Escalona, Zwick, et al., 2017; Seago et al., 2011). Considering the limited taxa sampling in the present study, sequencing more coccinellid mitogenomes covering more subfamilies/tribes and genera will be necessary to improve our understanding of mitogenomic phylogeny in Coccinellidae.

### 4.3 Evolution of mitochondrial protein‐coding genes in Coccinellidae

Generally, different mitochondrial genes evolve at different rates (Cheng, Chan, Zhang, Sun, & Sha, 2018; Yuan, Wei, Wang, Dou, & Wang, 2010; Yuan et al., 2015a, 2018). This was seen to be the case for the 13 mitochondrial PCGs of ladybirds, where *cox1*‐*3* and *cob* evolved the slowest and *atp8* the fastest. Site model results showed that the 13 coccinellid PCGs globally evolved under negative selection, with a relatively small percentage of codons evolving under neutral selection, reinforcing their crucial and conserved role for energy production. This result was also in agreement with the general trend of mitogenome evolution in animals (Almeida et al., 2015; da Fonseca et al., 2008; Yuan et al., 2018). For the concatenated PCGs, a significant difference in evolutionary rate was found among ladybirds with different diets (Table 2). However, not all of the 13 PCGs consistently showed the highest evolutionary rates within any one of the dietary groups. In contrast, a single gene, specific to one of three dietary groups, was seen to accumulate nonsynonymous mutations at the highest rate. Therefore, further studies using population genetics are needed to determine whether these mutations are due to random drift or to natural selection based on dietary adaptation.

A total of 40 codon sites in 10 PCGs showed signs of positive selection, as identified by CodeML and MEME models (Figure 4). However, most identified sites were presented in tip branches and/or dispersed throughout the whole tree, whereas only eight sites in six genes (*atp6*, 25, 74, and 122; *cox1*, 402; *cox3*, 70; *nad3*, 27; *nad4L*, 29; and *nad5*, 342) were potentially associated to dietary shifts/niche adaptation. Among these six PCGs, only *cox3* presented signals of positive selection in predatory ladybirds (branches s and v in Figure S1) and thus might be associated with dietary shifts. Several studies have detected non‐neutral changes in *cox3*, which encodes one key subunit of the cytochrome c oxidase complex (IV). For example, adaptive change of one amino acid site in *cox3* contributed to the high‐altitude adaptation in bar‐headed goose (Scott et al., 2011); substitutions in *cox1* and *cox3* from the freshwater fish *Poecilia* spp. were involved in the adaptation to H_2_S‐rich environments (Pfenninger et al., 2014). Against this background, it therefore seems plausible that *cox3* may be an important candidate gene associated with adaptation to dietary shifts in ladybirds.

Among the remaining five PCGs, three genes (*atp6*, *cox1*, and *nad5*) showed signs of positive selection in the branch m (Figure S1), whereas *nad3* and *nad4L* showed positive selection in branches q and p, respectively. ATP6, a core subunit of the F_0_F_1_‐ATP synthase (Complex V), is directly involved in ATP synthesis (Stock, Leslie, & Walker, 1999; Yoshida, Muneyuki, & Hisabori, 2001). Adaptive changes in *atp6* have been found to be associated with environmental and metabolic adaptation in animals (Escalona, Weadick, et al., 2017; Gu et al., 2012; Peng et al., 2012). For example, different feeding styles can influence adaptive evolution of ATP6 between blue sheep (*Pseudois*) living in different altitude environments (Peng et al., 2012). By contrast, *cox1* is generally regarded as being subject to neutral selection and thus has been extensively used in the studies of species identification, population genetics, and phylogeography in insects (Hebert, Ratnasingham, & Waard, 2003; Jinbo, Kato, & Ito, 2011). However, non‐neutral evolution of *cox1* has been reported in animals, such as mirids (Wang et al., 2017), the plateau pika (*Ochotona curzoniae*) (Luo et al., 2013), and freshwater fish (*Poecilia* spp.) (Pfenninger et al., 2014). Complex I (NADH dehydrogenase) is the first and largest of the five enzyme complexes that constitute the OXPHOS pathway (Ballard & Whitlock, 2004). ND5 is a conserved and highly hydrophobic protein, and positive selection at *nad5* has been suggested in several animals, such as the Tibetan wild ass (*Equus kiang*) (Luo et al., 2013), the plateau pika (*O. curzoniae*) (Luo et al., 2013), and various cephalopods (Almeida et al., 2015). Despite the importance of their function, ND3 and ND4L are components of the most smallest subunit of Complex I. Although adaptive evolution of ND3 and ND4L has rarely been observed in previous studies, their functional importance in the OXPHOS pathway suggests that these two PCGs may potentially be the target of positive selection. However, none of these three PCGs were present in the ancestor branch leading to each of three dietary types, indicating that potential positive selection in the three branches (m, p, and q in Figure S1) was not directly related to dietary shifts of ladybirds. Different ladybird species with the same type of diet can exhibit distinct dietary ranges and preferences (Escalona, Zwick, et al., 2017), which can significantly influence their life history traits and environmental adaptation. Even for the same species, differences in growth, reproduction, and metabolism were also obvious among individuals when feeding on different types of prey (Li et al., 2016). The branch m represents the common ancestral branch of two *Henosepilachna* species. Although the *Henosepilachna* and *Subcoccinella* genera are both herbivorous, the former mainly feed on family Solanaceae, while the latter mainly feed on Caryophyllaceae (Richards, Pope, & Eastop, 2010; Wang & Tu, 2005). Both *Anisosticta* and *Cheilomenes* feed primarily on aphids, and the former also feeds on Erysiphales fungi on Gramineae and Compositae flowering plants (Lablokoff‐Khnzorian, 1982). The genus *Coccinella* is significantly different from *Anisosticta* and *Cheilomenes* in feeding habits. *Coccinella* is mainly feeds on the larvae of Thysanoptera, Aleyrodidae, Psyllidae, and Cicadellidae, and the eggs and larvae of some butterflies and beetles (Lopatin, 1977). Therefore, the PCGs found to be under positive selection, specific to these branches, might be associated with their highly specialized diets. Furthermore, positive selection in these branches may be associated with other life history traits (e.g., generation time, body size, and behavior) (da Fonseca et al., 2008; Mitterboeck & Adamowicz, 2013; Saclier et al., 2018; Thomas, Welch, Lanfear, & Bromham, 2010); however, disentangling these effects is difficult.

In this study, the relationship between mitogenome evolution and diet in ladybirds was explored for the first time. Species sampling covered all three types of coccinellid diets, but the number of sequenced taxa was still quite limited compared to the richness of species within Coccinellidae. Due to the fact that most coccinellids were missing (only three tribes of approximately 40 total were included in the current study), our phylogeny might be not representative of Coccinellidae, and this may have distorted the observed results. Our results supported a potential association between adaptive evolution of *cox3* and coccinellid dietary shifts, but further functional testing is essential. For the remaining five PCGs under positive selection, their functional importance in these specific coccinellid groups deserves further evolutionary analyses. In addition, dietary shifts are associated with metabolic adaptation in animals, which manifests in genetic variations of both mitochondrial DNA and nuclear genomes (Ballard & Youngson, 2015; Seward & Kelly, 2016). Future study that includes more taxa representing different tribes including species with similar and contrasting diets and investigation of the sequence evolution of diet‐related nuclear genes will provide new insights into the molecular mechanism of adaptation to different diets in ladybirds.

## CONFLICT OF INTEREST

None declared.

## AUTHOR CONTRIBUTIONS

MLY designed the research. LJZ, QLZ, LZ, ML, XTW, and RQF performed the molecular experiments and analyzed the data. MLY and PAT contributed reagents/materials/analysis tools. MLY wrote the manuscript. PAT revised the manuscript. All authors read and approved the final manuscript.

## Data Availability

The mitogenomes of three coccinellid species newly sequenced in this study have been deposited in GenBank under accession numbers MG584726–28.
