# Supplementary figures and images for: Mitogenome evolution in ladybirds: Potential association with dietary adaptation
Source: Ecol Evol. 2020 Jan 2;10(2):1042–53. doi: 10.1002/ece3.5971 (PMC6988538; doi:10.1002/ece3.5971)

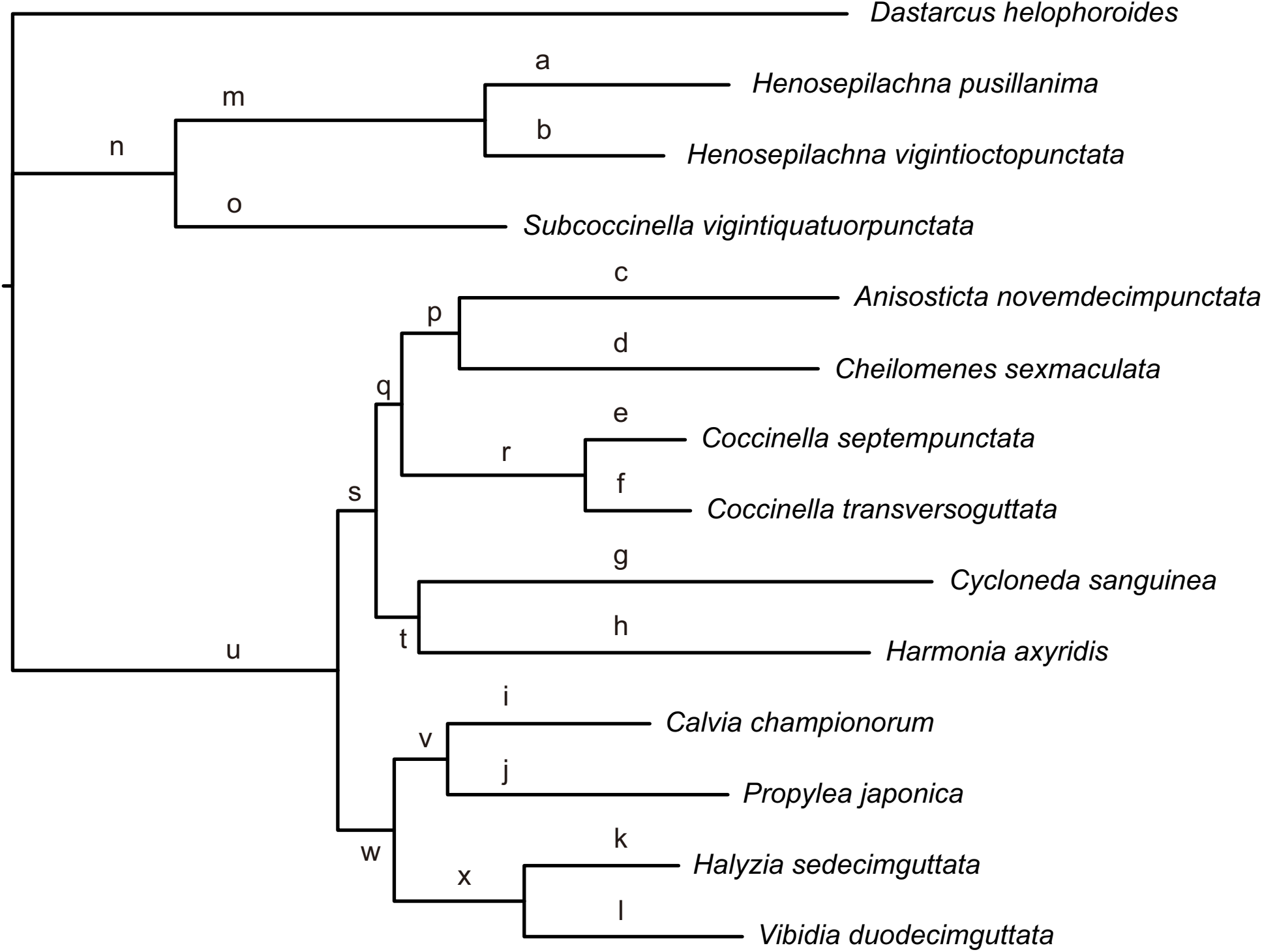

Supplement: Supplementary file 1 [file ECE3-10-1042-s001.pdf]

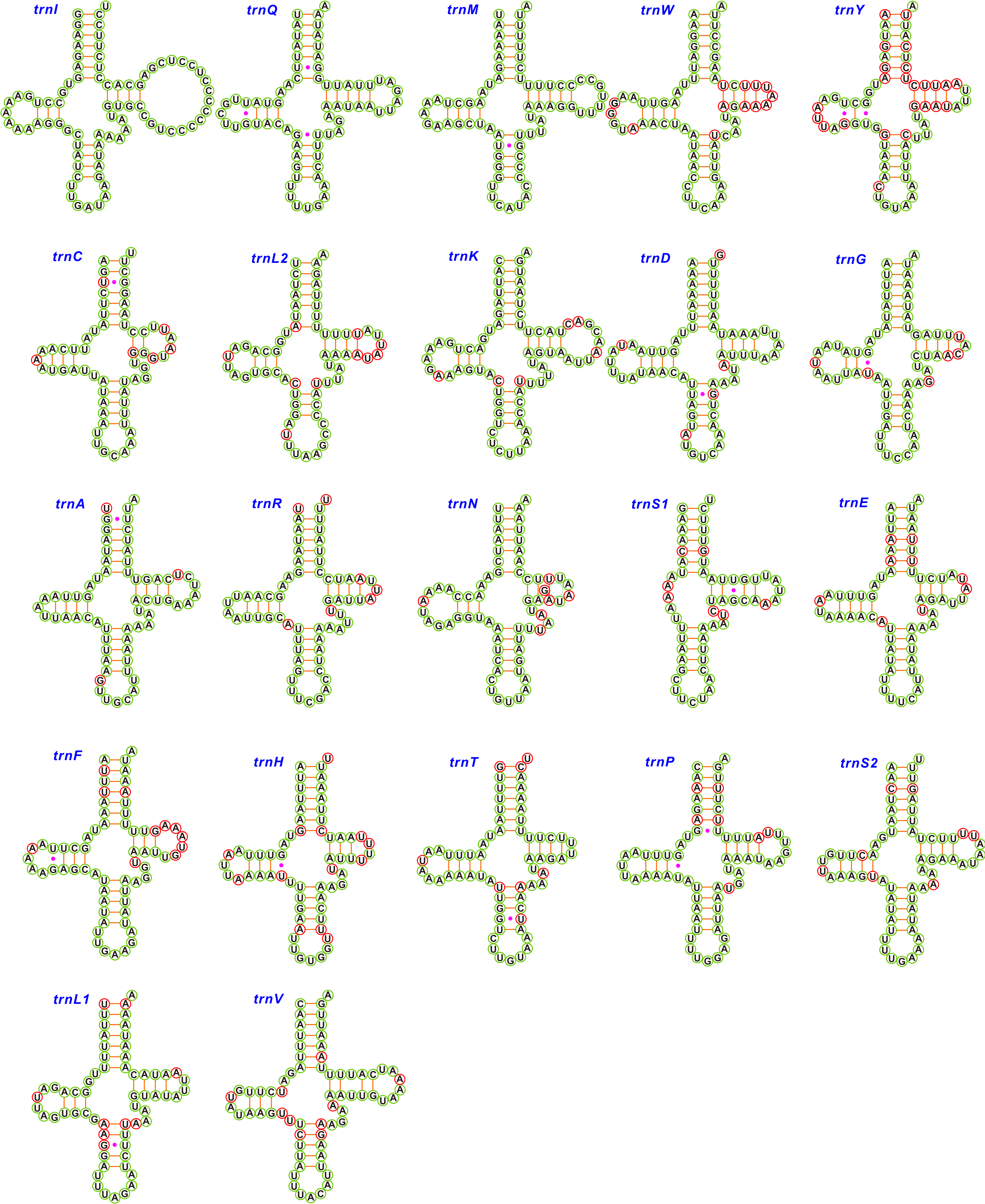

Supplement: Supplementary file 2 [file ECE3-10-1042-s002.pdf]
