# Supplementary material for: Mitogenome evolution in ladybirds: Potential association with dietary adaptation
Source: Ecol Evol. 2020 Jan 2;10(2):1042–53. doi: 10.1002/ece3.5971 (PMC6988538; doi:10.1002/ece3.5971)
Supplement: Supplementary file 3 [file ECE3-10-1042-s003.docx]

**Table S1** Sampling information.

| Species | Sampling locality | Sampling time |
| --- | --- | --- |
| *Coccinella transversoguttata* | Xifeng District, Qingyang City, Gansu Province, China | July 2014 |
| *Henosepilachna vigintioctopunctata* | Yinhai District, Beihai City, Guangxi Autonomous Region, China | August 2015 |
| *Vibidia duodecimguttata* | Xichuan County, Nanyang City, Henan Province, China | August 2015 |
