# Supplementary material for: Mitogenome evolution in ladybirds: Potential association with dietary adaptation
Source: Ecol Evol. 2020 Jan 2;10(2):1042–53. doi: 10.1002/ece3.5971 (PMC6988538; doi:10.1002/ece3.5971)
Supplement: Supplementary file 6 [file ECE3-10-1042-s006.docx]

**Table S4** Codon usage for the 13 mitochondrial protein-coding genes of *Coccinella transversoguttata*. RSCU, relative synonymous codon frequencies.

| Codon | Count | RSCU | Codon | Count | RSCU |
| --- | --- | --- | --- | --- | --- |
| UUU(F) | 343 | 1.8 | GCG(A) | 2 | 0.06 |
| UUC(F) | 38 | 0.2 | UAU(Y) | 154 | 1.72 |
| UUA(L) | 402 | 4.39 | UAC(Y) | 25 | 0.28 |
| UUG(L) | 37 | 0.4 | CAU(H) | 51 | 1.44 |
| CUU(L) | 39 | 0.43 | CAC(H) | 20 | 0.56 |
| CUC(L) | 6 | 0.07 | CAA(Q) | 49 | 1.92 |
| CUA(L) | 63 | 0.69 | CAG(Q) | 2 | 0.08 |
| CUG(L) | 2 | 0.02 | AAU(N) | 198 | 1.74 |
| AUU(I) | 344 | 1.76 | AAC(N) | 30 | 0.26 |
| AUC(I) | 47 | 0.24 | AAA(K) | 111 | 1.73 |
| AUA(M) | 269 | 1.78 | AAG(K) | 17 | 0.27 |
| AUG(M) | 33 | 0.22 | GAU(D) | 60 | 1.82 |
| GUU(V) | 64 | 1.72 | GAC(D) | 6 | 0.18 |
| GUC(V) | 0 | 0 | GAA(E) | 67 | 1.72 |
| GUA(V) | 76 | 2.04 | GAG(E) | 11 | 0.28 |
| GUG(V) | 9 | 0.24 | UGU(C) | 23 | 1.7 |
| UCU(S) | 109 | 2.67 | UGC(C) | 4 | 0.3 |
| UCC(S) | 18 | 0.44 | UGA(W) | 85 | 1.91 |
| UCA(S) | 79 | 1.94 | UGG(W) | 4 | 0.09 |
| UCG(S) | 7 | 0.17 | CGU(R) | 18 | 1.36 |
| CCU(P) | 88 | 2.86 | CGC(R) | 2 | 0.15 |
| CCC(P) | 9 | 0.29 | CGA(R) | 31 | 2.34 |
| CCA(P) | 23 | 0.75 | CGG(R) | 2 | 0.15 |
| CCG(P) | 3 | 0.1 | AGU(S) | 22 | 0.54 |
| ACU(T) | 95 | 2.25 | AGC(S) | 2 | 0.05 |
| ACC(T) | 14 | 0.33 | AGA(S) | 87 | 2.13 |
| ACA(T) | 56 | 1.33 | AGG(S) | 2 | 0.05 |
| ACG(T) | 4 | 0.09 | GGU(G) | 42 | 0.94 |
| GCU(A) | 82 | 2.47 | GGC(G) | 9 | 0.2 |
| GCC(A) | 16 | 0.48 | GGA(G) | 100 | 2.23 |
| GCA(A) | 33 | 0.99 | GGG(G) | 28 | 0.63 |
