# Supplementary material for: Mitogenome evolution in ladybirds: Potential association with dietary adaptation
Source: Ecol Evol. 2020 Jan 2;10(2):1042–53. doi: 10.1002/ece3.5971 (PMC6988538; doi:10.1002/ece3.5971)
Supplement: Supplementary file 7 [file ECE3-10-1042-s007.docx]

**Table S5** Codon usage for the 13 mitochondrial protein-coding genes of *Vibidia duodecimguttata*. RSCU, relative synonymous codon frequencies.

| Codon | Count | RSCU | Codon | Count | RSCU |
| --- | --- | --- | --- | --- | --- |
| UUU(F) | 326 | 1.75 | GCG(A) | 6 | 0.17 |
| UUC(F) | 47 | 0.25 | UAU(Y) | 133 | 1.59 |
| UUA(L) | 420 | 4.48 | UAC(Y) | 34 | 0.41 |
| UUG(L) | 37 | 0.4 | CAU(H) | 57 | 1.52 |
| CUU(L) | 39 | 0.42 | CAC(H) | 18 | 0.48 |
| CUC(L) | 5 | 0.05 | CAA(Q) | 47 | 1.68 |
| CUA(L) | 57 | 0.61 | CAG(Q) | 9 | 0.32 |
| CUG(L) | 4 | 0.04 | AAU(N) | 180 | 1.67 |
| AUU(I) | 350 | 1.79 | AAC(N) | 36 | 0.33 |
| AUC(I) | 42 | 0.21 | AAA(K) | 105 | 1.75 |
| AUA(M) | 268 | 1.81 | AAG(K) | 15 | 0.25 |
| AUG(M) | 28 | 0.19 | GAU(D) | 50 | 1.52 |
| GUU(V) | 58 | 1.52 | GAC(D) | 16 | 0.48 |
| GUC(V) | 4 | 0.1 | GAA(E) | 64 | 1.66 |
| GUA(V) | 83 | 2.17 | GAG(E) | 13 | 0.34 |
| GUG(V) | 8 | 0.21 | UGU(C) | 16 | 1.28 |
| UCU(S) | 118 | 2.79 | UGC(C) | 9 | 0.72 |
| UCC(S) | 8 | 0.19 | UGA(W) | 82 | 1.86 |
| UCA(S) | 81 | 1.92 | UGG(W) | 6 | 0.14 |
| UCG(S) | 8 | 0.19 | CGU(R) | 18 | 1.38 |
| CCU(P) | 75 | 2.46 | CGC(R) | 2 | 0.15 |
| CCC(P) | 18 | 0.59 | CGA(R) | 27 | 2.08 |
| CCA(P) | 26 | 0.85 | CGG(R) | 5 | 0.38 |
| CCG(P) | 3 | 0.1 | AGU(S) | 29 | 0.69 |
| ACU(T) | 85 | 2.06 | AGC(S) | 8 | 0.19 |
| ACC(T) | 19 | 0.46 | AGA(S) | 74 | 1.75 |
| ACA(T) | 60 | 1.45 | AGG(S) | 12 | 0.28 |
| ACG(T) | 1 | 0.02 | GGU(G) | 34 | 0.72 |
| GCU(A) | 74 | 2.06 | GGC(G) | 9 | 0.19 |
| GCC(A) | 12 | 0.33 | GGA(G) | 111 | 2.34 |
| GCA(A) | 52 | 1.44 | GGG(G) | 36 | 0.76 |
