# Supplementary material for: Mitogenome evolution in ladybirds: Potential association with dietary adaptation
Source: Ecol Evol. 2020 Jan 2;10(2):1042–53. doi: 10.1002/ece3.5971 (PMC6988538; doi:10.1002/ece3.5971)
Supplement: Supplementary file 8 [file ECE3-10-1042-s008.docx]

**Table S6** Codon usage for the 13 mitochondrial protein-coding genes of *Henosepilachna vigintioctopunctata*. RSCU, relative synonymous codon frequencies.

| Codon | Count | RSCU | Codon | Count | RSCU |
| --- | --- | --- | --- | --- | --- |
| UUU(F) | 375 | 1.82 | GCG(A) | 1 | 0.03 |
| UUC(F) | 38 | 0.18 | UAU(Y) | 124 | 1.68 |
| UUA(L) | 482 | 4.79 | UAC(Y) | 24 | 0.32 |
| UUG(L) | 29 | 0.29 | CAU(H) | 56 | 1.72 |
| CUU(L) | 58 | 0.58 | CAC(H) | 9 | 0.28 |
| CUC(L) | 4 | 0.04 | CAA(Q) | 61 | 1.94 |
| CUA(L) | 30 | 0.3 | CAG(Q) | 2 | 0.06 |
| CUG(L) | 1 | 0.01 | AAU(N) | 187 | 1.8 |
| AUU(I) | 377 | 1.89 | AAC(N) | 21 | 0.2 |
| AUC(I) | 21 | 0.11 | AAA(K) | 118 | 1.79 |
| AUA(M) | 239 | 1.8 | AAG(K) | 14 | 0.21 |
| AUG(M) | 27 | 0.2 | GAU(D) | 54 | 1.74 |
| GUU(V) | 60 | 1.82 | GAC(D) | 8 | 0.26 |
| GUC(V) | 1 | 0.03 | GAA(E) | 76 | 1.88 |
| GUA(V) | 64 | 1.94 | GAG(E) | 5 | 0.12 |
| GUG(V) | 7 | 0.21 | UGU(C) | 20 | 1.74 |
| UCU(S) | 110 | 2.4 | UGC(C) | 3 | 0.26 |
| UCC(S) | 17 | 0.37 | UGA(W) | 79 | 1.7 |
| UCA(S) | 93 | 2.03 | UGG(W) | 14 | 0.3 |
| UCG(S) | 3 | 0.07 | CGU(R) | 15 | 1.15 |
| CCU(P) | 77 | 2.46 | CGC(R) | 2 | 0.15 |
| CCC(P) | 18 | 0.58 | CGA(R) | 34 | 2.62 |
| CCA(P) | 30 | 0.96 | CGG(R) | 1 | 0.08 |
| CCG(P) | 0 | 0 | AGU(S) | 31 | 0.68 |
| ACU(T) | 79 | 2.26 | AGC(S) | 1 | 0.02 |
| ACC(T) | 12 | 0.34 | AGA(S) | 110 | 2.4 |
| ACA(T) | 48 | 1.37 | AGG(S) | 1 | 0.02 |
| ACG(T) | 1 | 0.03 | GGU(G) | 48 | 1.05 |
| GCU(A) | 67 | 2.25 | GGC(G) | 10 | 0.22 |
| GCC(A) | 14 | 0.47 | GGA(G) | 103 | 2.25 |
| GCA(A) | 37 | 1.24 | GGG(G) | 22 | 0.48 |
