# Supplementary material for: Mitogenome evolution in ladybirds: Potential association with dietary adaptation
Source: Ecol Evol. 2020 Jan 2;10(2):1042–53. doi: 10.1002/ece3.5971 (PMC6988538; doi:10.1002/ece3.5971)
Supplement: Supplementary file 9 [file ECE3-10-1042-s009.docx]

**Table S7** The effective number of codons (ENC) and G+C content of all positions (GCa) and the third positions (GC3).

| Subfamily | Tribe | Speceis | ENC | GC3 | GCa |
| --- | --- | --- | --- | --- | --- |
| Coccidulinae | Coccinellini | *Coccinella septempunctata* | 34.38 | 0.10 | 0.23 |
|  |  | *Anisosticta novemdecimpunctata* | 34.09 | 0.09 | 0.22 |
|  |  | *Calvia championorum* | 36.24 | 0.11 | 0.23 |
|  |  | *Cheilomenes sexmaculata* | 35.28 | 0.11 | 0.23 |
|  |  | *Coccinella transversoguttata* | 34.31 | 0.11 | 0.23 |
|  |  | *Cycloneda sanguinea* | 34.91 | 0.14 | 0.23 |
|  |  | *Harmonia axyridis* | 34.61 | 0.10 | 0.23 |
|  |  | *Propylea japonica* | 33.75 | 0.09 | 0.21 |
|  | Halyziini | *Halyzia sedecimguttata* | 36.13 | 0.12 | 0.23 |
|  |  | *Vibidia duodecimguttata* | 37.08 | 0.13 | 0.24 |
| Epilachninae | Epilachnini | *Henosepilachna pusillanima* | 36.62 | 0.12 | 0.23 |
|  |  | *Henosepilachna vigintioctopunctata* | 33.80 | 0.09 | 0.22 |
|  |  | *Subcoccinella vigintiquatuorpunctata* | 36.79 | 0.14 | 0.25 |
