# Supplementary material for: Mitogenome evolution in ladybirds: Potential association with dietary adaptation
Source: Ecol Evol. 2020 Jan 2;10(2):1042–53. doi: 10.1002/ece3.5971 (PMC6988538; doi:10.1002/ece3.5971)
Supplement: Supplementary file 10 [file ECE3-10-1042-s010.docx]

**Table S8** Saturation test implemented in DAMBE.

| Gene regions | Symmetrical tree | | | Asymmetrical tree | | |
| --- | --- | --- | --- | --- | --- | --- |
|  | *Iss* | *Iss.c* | *P* | | *Iss.c* | *P* |
| all positions of 13 PCGs | 0.3705 | 0.8382 | < 0.0001 | | 0.6828 | < 0.0001 |
| 1st positions of 13 PCGs | 0.3196 | 0.8222 | < 0.0001 | | 0.6779 | < 0.0001 |
| 2nd positions of 13 PCGs | 0.1922 | 0.8222 | < 0.0001 | | 0.6779 | < 0.0001 |
| 3rd positions of 13 PCGs | 0.6958 | 0.8222 | < 0.0001 | | 0.6779 | 0.0568 |
