# Supplementary material for: Mitogenome evolution in ladybirds: Potential association with dietary adaptation
Source: Ecol Evol. 2020 Jan 2;10(2):1042–53. doi: 10.1002/ece3.5971 (PMC6988538; doi:10.1002/ece3.5971)
Supplement: Supplementary file 11 [file ECE3-10-1042-s011.docx]

**Table S9** The best partitioning schemes and substitution models selected by PartitionFinder for the P123 and P12 datasets.

| Data matrix | Subset | Best-fit scheme | Model |
| --- | --- | --- | --- |
| P123 | P1 | *atp6_*pos1*, cob_*pos1*, cox1_*pos1*, cox2_*pos1*, cox3_*pos1*, nad3_*pos1 | GTR+I+G |
|  | P2 | *atp6_*pos2*, cob_*pos2*, cox1_*pos2*, cox2_*pos2*, cox3_*pos2*, nad1_*pos2*, nad2_*pos2*, nad3_*pos2*, nad4L_*pos2*, nad4_*pos2*, nad5_*pos2*, nad6_*pos2 | GTR+I+G |
|  | P3 | *atp6_*pos3*, atp8_*pos3*, cob_*pos3*, cox1_*pos3*, cox2_*pos3*, cox3_*pos3*, nad2_*pos3*, nad3_*pos3*, nad6_*pos3 | GTR+I+G |
|  | P4 | *atp8_*pos1*, atp8_*pos2*, nad1_*pos1*, nad2_*pos1*, nad4L_*pos1*, nad4_*pos1*, nad5_*pos1*, nad6_*pos1 | GTR+I+G |
|  | P5 | *nad1_*pos3*, nad4L_*pos3*, nad4_*pos3*, nad5_*pos3 | HKY+G |
| P12 | P1 | *atp6_*pos1*, cob_*pos1*, cox1_*pos1*, cox2_*pos1*, cox3_*pos1*, nad3_*pos1 | GTR+I+G |
|  | P2 | *atp8_*pos1*, atp8_*pos2*, nad2_*pos1*, nad6_*pos1 | GTR+I+G |
|  | P3 | *nad1_*pos1*, nad4L_*pos1*, nad4_*pos1*, nad5_*pos1 | GTR+I+G |
|  | P4 | *atp6_*pos2*, cob_*pos2*, cox1_*pos2*, cox2_*pos2*, cox3_*pos2*, nad1_*pos2*, nad2_*pos2*, nad3_*pos2*, nad4L_*pos2*, nad4_*pos2*, nad5_*pos2*, nad6_*pos2 | GTR+I+G |
