# Supplementary material for: Mitogenome evolution in ladybirds: Potential association with dietary adaptation
Source: Ecol Evol. 2020 Jan 2;10(2):1042–53. doi: 10.1002/ece3.5971 (PMC6988538; doi:10.1002/ece3.5971)
Supplement: Supplementary file 12 [file ECE3-10-1042-s012.docx]

**Table S10** Site-specific ML parameter estimates for each of the 13 mitochondrial protein-coding genes in 13 coccinellid species.

| Gene | Model | Parameters | lnL | Model test | LRT | *P* | Positively selected sites |
| --- | --- | --- | --- | --- | --- | --- | --- |
| *atp6* | M0: one-ratio | *ω* = 0.02608 | -4887.8969 |  |  |  |  |
|  | M1a: neutral | p0 = 0.96448, p1 = 0.03552, ω0 = 0.02889, ω1 = 1.00000 | -4875.9894 | M1a vs M0 | 23.82 | 0 |  |
|  | M2a: selection | p0 = 0.96448, p1 = 0.00126, p2 = 0.03425, ω0 = 0.02889, ω1 = 1, ω2 = 1.00000 | -4875.9894 | M2a vs M1a | 0 | 1 | None |
|  | M3: discrete | ω0 = 0.00008, p0 = 0.40970, ω1 = 0.01920 , p1 = 0.31665, ω2 = 0.08768, p2 = 0.27365 | -4696.3608 | M3 vs M0 | 383 | 0 |  |
|  | M7: beta | p = 0.36117, q = 11.14238 | -4700.9499 |  |  |  |  |
|  | M8: beta & *ω* | p0 = 0.99999, p1 = 0.00001, p = 0.36117, q =11.14236, ω = 1.00000 | -4700.9520 | M8 vs M7 | 0 | 0.99 | None |
| *atp8* | M0: one-ratio | *ω* = 0.00890 | -1528.0911 |  |  |  |  |
|  | M1a: neutral | p0 = 0.60036, p1 = 0.39964, ω0 = 0.07069, ω1 = 1 | -1501.5707 | M1a vs M0 | 53.04 | 0 |  |
|  | M2a: selection | p0 = 0.60036, p1 = 0.16300, p2 = 0.23664, ω0 = 0.07069, ω1 = 1.00000, ω2 = 1.00000 | -1501.5707 | M2a vs M1a | 0 | 1 | None |
|  | M3: discrete | ω0 = 0.00000, p0 = 0.12181, ω1 = 0.00424, p1 = 0.47339, ω2 = 0.02751, p2 = 0.40479 | -1464.7617 | M3 vs M0 | 127 | 0 |  |
|  | M7: beta | p = 0.67416, q = 43.84654 | -1468.0015 |  |  |  |  |
|  | M8: beta & *ω* | p0 = 0.99999, p1 = 0.00001, p = 0.67416, q = 43.84684, ω = 1.00000 | -1468.0018 | M8 vs M7 | 0.00 | 0.99 | None |
| *cob* | M0: one-ratio | *ω* = 0.01823 | -8009.9838 |  |  |  |  |
|  | M1a: neutral | p0 = 0.97843, p1 = 0.02157, ω0 = 0.01714, ω1 = 1 | -7990.8833 | M1a vs M0 | 38.20 | 0 |  |
|  | M2a: selection | p0 = 0.97843, p1 = 0.02157, p2 = 0.00000, ω0 = 0.01714, ω1 = 1.00000, ω2 = 43.34227 | -7990.8833 | M2a vs M1a | 0 | 1 | None |
|  | M3: discrete | ω0 = 0.00130, p0 = 0.55831, ω1 = 0.02791, p1 = 0.30667, ω2 = 0.07423, p2 = 0.13502 | -7746.9058 | M3 vs M0 | 526 | 0 |  |
|  | M7: beta | p = 0.36089, q = 17.38439 | -7749.9932 |  |  |  |  |
|  | M8: beta & *ω* | p0 = 0.99999, p1 = 0.00001, p = 0.36089, q = 17.38454, ω = 1.00000 | -7749.9968 | M8 vs M7 | 0 | 0.99 | None |
| *cox1* | M0: one-ratio | *ω* = 0.01364 | -9463.1435 |  |  |  |  |
|  | M1a: neutral | p0 = 0.97019, p1 = 0.02981, ω0 = 0.01161, ω1 = 1 | -9403.1227 | M1a vs M0 | 120.04 | 0 |  |
|  | M2a: selection | p0 = 0.97019, p1 = 0.02981, p2 = 0.00000, ω0 = 0.01161, ω1 = 1, ω2 = 70.99259 | -9403.1227 | M2a vs M1a | 0 | 1 | None |
|  | M3: discrete | ω0 = 0.00123, p0 = 0.74348, ω1 = 0.03230, p1 = 0.19067, ω2 = 0.11940, p2 = 0.06585 | -9142.2963 | M3 vs M0 | 642 | 0 |  |
|  | M7: beta | p = 0.16260, q = 8.77132 | -9142.9231 |  |  |  |  |
|  | M8: beta & *ω* | p0 = 0.99899, p1 = 0.00101, p = 0.16367, q = 9.03857, ω = 1.00000 | -9142.8442 | M8 vs M7 | 0.16 | 0.92 | None |
| *cox2* | M0: one-ratio | *ω* = 0.01740 | -4612.7036 |  |  |  |  |
|  | M1a: neutral | p0 = 0.97319, p1 = 0.02681, ω0 = 0.01623, ω1 = 1 | -4591.5266 | M1a vs M0 | 42.35 | 0 |  |
|  | M2a: selection | p0 = 0.97319, p1 = 0.02681, p2 = 0.00000, ω0 = 0.01623, ω1 = 1, ω2 = 21.12806 | -4591.5266 | M2a vs M1a | 0 | 1 | None |
|  | M3: discrete | ω0 = 0.00202, p0 = 0.58705, ω1 = 0.02817, p1 = 0.34494, ω2 = 0.10667, p2 = 0.06801 | -4480.9515 | M3 vs M0 | 264 | 0 |  |
|  | M7: beta | p = 0.37251, q = 18.07670 | -4483.4730 |  |  |  |  |
|  | M8: beta & *ω* | p0 = 0.99567, p1 = 0.00433, p = 0.39262, q = 20.88774, ω = 1.00000 | -4481.8063 | M8 vs M7 | 3 | 0.18 | None |
| *cox3* | M0: one-ratio | *ω* = 0.03195 | -5760.1929 |  |  |  |  |
|  | M1a: neutral | p0 = 0.92814, p1 = 0.07186, ω0 = 0.02891, ω1 = 1 | -5691.1451 | M1a vs M0 | 138.10 | 0 |  |
|  | M2a: selection | p0 = 0.92814, p1 = 0.04797, p2 = 0.02389, ω0 = 0.02891, ω1 = 1.00000, ω2 = 1.00000 | -5691.1451 | M2a vs M1a | 0 | 1 | None |
|  | M3: discrete | ω0 = 0.00050, p0 = 0.49701, ω1 = 0.03063, p1 = 0.30862, ω2 = 0.14530, p2 = 0.19438 | -5485.8343 | M3 vs M0 | 549 | 0 |  |
|  | M7: beta | p = 0.26549, q = 6.28958 | -5487.3350 |  |  |  |  |
|  | M8: beta & *ω* | p0 = 0.99999, p1 = 0.00001, p = 0.26549, q = 6.28961, ω = 2.78954 | -5487.3374 | M8 vs M7 | 0 | 0.99 | None |
| *nad1* | M0: one-ratio | *ω* = 0.01820 | -6183.4994 |  |  |  |  |
|  | M1a: neutral | p0 = 0.97424, p1 = 0.02576, ω0 = 0.01787, ω1 = 1 | -6156.2321 | M1a vs M0 | 54.53 | 0 |  |
|  | M2a: selection | p0 = 0.97424, p1 = 0.02576, p2 = 0.00000, ω0 = 0.01787, ω1 = 1, ω2 = 17.53691 | -6156.2321 | M2a vs M1a | 0 | 1 | None |
|  | M3: discrete | ω0 = 0.00188, p0 = 0.52786, ω1 = 0.03185, p1 = 0.38642, ω2 = 0.09675, p2 = 0.08571 | -5969.4998 | M3 vs M0 | 428 | 0 |  |
|  | M7: beta | p = 0.43235, q = 18.30911 | -5973.8092 |  |  |  |  |
|  | M8: beta & *ω* | p0 = 0.99999, p1 = 0.00001, p = 0.42863, q = 18.05823, ω = 28.32026 | -5973.8174 | M8 vs M7 | 0 | 0.99 | None |
| *nad2* | M0: one-ratio | *ω* = 0.01825 | -9264.5911 |  |  |  |  |
|  | M1a: neutral | p0 = 0.96596, p1 = 0.03404, ω0 = 0.01674, ω1 = 1 | -9218.6611 | M1a vs M0 | 91.86 | 0 |  |
|  | M2a: selection | p0 = 0.96596, p1 = 0.00651, p2 = 0.02753, ω0 = 0.01674, ω1 = 1, ω2 = 1.00000 | -9218.6611 | M2a vs M1a | 0 | 1 | None |
|  | M3: discrete | ω0 = 0.00131, p0 = 0.22929, ω1 = 0.01446, p1 = 0.55282, ω2 = 0.05315, p2 = 0.21789 | -9036.4726 | M3 vs M0 | 456 | 0 |  |
|  | M7: beta | p = 0.85892, q = 39.88646 | -9033.5318 |  |  |  |  |
|  | M8: beta & *ω* | p0 = 0.99428, p1 = 0.00572, p = 0.91080, q = 45.48374, ω = 1.00000 | -9029.1444 | M8 vs M7 | 8.77 | 0.01 | None |
| *nad3* | M0: one-ratio | *ω* = 0.02021 | -2944.5302 |  |  |  |  |
|  | M1a: neutral | p0 = 0.91999, p1 = 0.08001, ω0 = 0.01558, ω1 = 1 | -2919.4877 | M1a vs M0 | 50.09 | 0 |  |
|  | M2a: selection | p0 = 0.91999, p1 = 0.00305, p2 = 0.07696, ω0 = 0.01558, ω1 = 1, ω2 =1.00000 | -2919.4877 | M2a vs M1a | 0 | 1 | None |
|  | M3: discrete | ω0 = 0.00068, p0 = 0.45334, ω1 = 0.01986, p1 = 0.27713, ω2 = 0.06761, p2 = 0.26953 | -2789.8367 | M3 vs M0 | 309 | 0 |  |
|  | M7: beta | p = 0.34717, q = 12.85833 | -2792.2516 |  |  |  |  |
|  | M8: beta & *ω* | p0 = 0.99999, p1 = 0.00001, p = 0.34717, q = 12.85880, ω = 18.17950 | -2792.2522 | M8 vs M7 | 0 | 0.99 | None |
| *nad4* | M0: one-ratio | *ω* = 0.01456 | -9795.2894 |  |  |  |  |
|  | M1a: neutral | p0 = 0.96558, p1 = 0.03442, ω0 = 0.01435, ω1 = 1 | -9729.5454 | M1a vs M0 | 131.49 | 0 |  |
|  | M2a: selection | p0 = 0.96559, p1 = 0.03441, p2 = 0.00000, ω0 = 0.01435, ω1 = 1, ω2 = 27.45074 | -9729.5454 | M2a vs M1a | 0 | 1 | None |
|  | M3: discrete | ω0 = 0.00160, p0 = 0.45269, ω1 = 0.02160, p1 = 0.45923, ω2 = 0.08079, p2 =0.08808 | -9431.8021 | M3 vs M0 | 727 | 0 |  |
|  | M7: beta | p = 0.51360, q = 25.96155 | -9434.0699 |  |  |  |  |
|  | M8: beta & *ω* | p0 = 0.99999, p1 = 0.00001, p = 0.51361, q = 25.96261, ω = 1.00000 | -9434.0727 | M8 vs M7 | 0.01 | 0.99 | None |
| *nad4L* | M0: one-ratio | *ω* = 0.02388 | -2152.3714 |  |  |  |  |
|  | M1a: neutral | p0 = 0.94208, p1 = 0.05792, ω0 = 0.02397, ω1 = 1 | -2123.4751 | M1a vs M0 | 57.79 | 0 |  |
|  | M2a: selection | p0 = 0.94208, p1 = 0.00041, p2 = 0.05751, ω0 = 0.02397, ω1 = 1, ω2 =1.00000 | -2123.4751 | M2a vs M1a | 0 | 1 | None |
|  | M3: discrete | ω0 = 0.00316, p0 = 0.44318, ω1 = 0.03228, p1 = 0.45149, ω2 = 0.14602, p2 = 0.10532 | -2067.5802 | M3 vs M0 | 170 | 0 |  |
|  | M7: beta | p = 0.51590, q = 14.89917 | -2066.9590 |  |  |  |  |
|  | M8: beta & *ω* | p0 = 0.98144, p1 = 0.01856, p = 0.58575, q = 19.92080, ω = 6.07442 | -2065.7276 | M8 vs M7 | 2 | 0.29 | 13M 0.956* |
| *nad5* | M0: one-ratio | *ω* = 0.01838 | -13740.6112 |  |  |  |  |
|  | M1a: neutral | p0 = 0.92657, p1 = 0.07343, ω0 = 0.01897, ω1 = 1 | -13556.0740 | M1a vs M0 | 369.07 | 0 |  |
|  | M2a: selection | p0 = 0.92657, p1 = 0.00004, p2 = 0.07339, ω0 = 0.01897, ω1 = 1, ω2 = 1.00000 | -13556.0740 | M2a vs M1a | 0 | 1 | None |
|  | M3: discrete | ω0 = 0.00257, p0 = 0.50288, ω1 = 0.02724, p1 = 0.36498, ω2 = 0.09216, p2 = 0.13214 | -13085.3844 | M3 vs M0 | 1310 | 0 |  |
|  | M7: beta | p = 0.43508, q = 15.99937 | -13071.1961 |  |  |  |  |
|  | M8: beta & *ω* | p0 = 0.99267, p1 = 0.00733, p = 0.45814, q = 18.04691, ω = 1.00000 | -13069.6819 | M8 vs M7 | 3.03 | 0.22 | None |
| *nad6* | M0: one-ratio | *ω* = 0.01360 | -4517.1813 |  |  |  |  |
|  | M1a: neutral | p0 = 0.91518, p1 = 0.08482, ω0 = 0.01298, ω1 = 1 | -4479.5825 | M1a vs M0 | 75.20 | 0 |  |
|  | M2a: selection | p0 = 0.91518, p1 = 0.02239, p2 = 0.06242, ω0 = 0.01298, ω1 = 1, ω2 = 1.00000 | -4479.5825 | M2a vs M1a | 0 | 1 | None |
|  | M3: discrete | ω0 = 0.00216, p0 = 0.38658, ω1 = 0.01872, p1 = 0.47992, ω2 = 0.06357, p2 = 0.13349 | -4364.4386 | M3 vs M0 | 305 | 0 |  |
|  | M7: beta | p = 0.69581, q = 35.35025 | -4363.8320 |  |  |  |  |
|  | M8: beta & *ω* | p0 = 0.99761, p1 = 0.00239, p = 0.70173, q = 36.04437, ω = 1.00000 | -4363.8198 | M8 vs M7 | 0 | 0.98 | None |
