# Supplementary material for: Mitogenome evolution in ladybirds: Potential association with dietary adaptation
Source: Ecol Evol. 2020 Jan 2;10(2):1042–53. doi: 10.1002/ece3.5971 (PMC6988538; doi:10.1002/ece3.5971)
Supplement: Supplementary file 13 [file ECE3-10-1042-s013.docx]

**Table S11** Results of the branch-site model in PAML.

| Forebranch | Gene | Model | Likelihood (*L*) | Site class | 0 | 1 | 2a | 2b | 2Δ*L* | *P* | Positively selected sites |
| --- | --- | --- | --- | --- | --- | --- | --- | --- | --- | --- | --- |
| s + v (predatory) | *cox3* | Model A (Null) | -5683.6 | proportion | 0.9067 | 0.0600 | 0.0312 | 0.0021 |  |  |  |
|  |  |  |  | background *ω* | 0.0280 | 1.0000 | 0.0280 | 1.0000 |  |  |  |
|  |  |  |  | foreground *ω* | 0.0280 | 1.0000 | 1.0000 | 1.0000 |  |  |  |
|  |  | Model A (Alternative) | -5691.1 | proportion | 0.9281 | 0.0719 | 0.0000 | 0.0000 |  |  |  |
|  |  |  |  | background *ω* | 0.0289 | 1.0000 | 0.0289 | 1.0000 |  |  |  |
|  |  |  |  | foreground *ω* | 0.0289 | 1.0000 | 67.2677 | 67.2677 | 14.97 | 0.000109 | 70E 0.99** |
| m | *atp6* | Model A (Null) | -4863.1 | proportion | 0.8615 | 0.0316 | 0.1031 | 0.0038 |  |  |  |
|  |  |  |  | background *ω* | 0.0267 | 1.0000 | 0.0267 | 1.0000 |  |  |  |
|  |  |  |  | foreground *ω* | 0.0267 | 1.0000 | 1.0000 | 1.0000 |  |  |  |
|  |  | Model A (Alternative) | -4859.7 | proportion | 0.8996 | 0.0326 | 0.0654 | 0.0024 |  |  |  |
|  |  |  |  | background *ω* | 0.0273 | 1.0000 | 0.0273 | 1.0000 |  |  |  |
|  |  |  |  | foreground *ω* | 0.0273 | 1.0000 | 0.0273 | 0.0273 | 6.930486 | 0.008474 | 25L 0.99*; 74S 0.98*; 122Y 0.98* |
| o | *cox3* | Model A (Null) | -5683.7 | proportion | 0.8919 | 0.0675 | 0.0377 | 0.0029 |  |  |  |
|  |  |  |  | background *ω* | 0.0275 | 1.0000 | 0.0275 | 1.0000 |  |  |  |
|  |  |  |  | foreground *ω* | 0.0275 | 1.0000 | 1.0000 | 1.0000 |  |  |  |
|  |  | Model A (Alternative) | -5680.5 | proportion | 0.9038 | 0.0702 | 0.0241 | 0.0019 |  |  |  |
|  |  |  |  | background *ω* | 0.0277 | 1.0000 | 0.0277 | 1.0000 |  |  |  |
|  |  |  |  | foreground *ω* | 0.0277 | 1.0000 | 30.0585 | 30.0585 | 6.342786 | 0.011786 | 26N 0.95*; 41N 0.98*; 217V 0.97* |
| o | *nad4L* | Model A (Null) | -2119.6 | proportion | 0.9119 | 0.0548 | 0.0315 | 0.0019 |  |  |  |
|  |  |  |  | background *ω* | 0.0224 | 1.0000 | 0.0224 | 1.0000 |  |  |  |
|  |  |  |  | foreground *ω* | 0.0224 | 1.0000 | 1.0000 | 1.0000 |  |  |  |
|  |  | Model A (Alternative) | -2117.6 | proportion | 0.9125 | 0.0538 | 0.0319 | 0.0019 |  |  |  |
|  |  |  |  | background *ω* | 0.0241 | 1.0000 | 0.0241 | 1.0000 |  |  |  |
|  |  |  |  | foreground *ω* | 0.0241 | 1.0000 | 22.1838 | 22.1838 | 3.981318 | 0.046008 | 98K 0.99** |
| h | *atp6* | Model A (Null) | -4866.3 | proportion | 0.8864 | 0.0345 | 0.0762 | 0.0030 |  |  |  |
|  |  |  |  | background *ω* | 0.0272 | 1.0000 | 0.0272 | 1.0000 |  |  |  |
|  |  |  |  | foreground *ω* | 0.0272 | 1.0000 | 1.0000 | 1.0000 |  |  |  |
|  |  | Model A (Alternative) | -4864.4 | proportion | 0.9031 | 0.0357 | 0.0589 | 0.0023 |  |  |  |
|  |  |  |  | background *ω* | 0.0278 | 1.0000 | 0.0278 | 1.0000 |  |  |  |
|  |  |  |  | foreground *ω* | 0.0278 | 1.0000 | 74.8175 | 74.8175 | 3.901206 | 0.048251 | 74S 0.95*; 94S 0.97* |
